# Notching early repolarization pattern in inferior leads increases risk of ventricular tachyarrhythmias in patients with acute myocardial infarction: a meta-analysis

**DOI:** 10.1038/srep15845

**Published:** 2015-11-02

**Authors:** Zhiwei Zhang, Konstantinos P. Letsas, Yajuan Yang, Panagiotis Korantzopoulos, Guangping Li, Gan-Xin Yan, Tong Liu

**Affiliations:** 1 Tianjin Key Laboratory of Ionic-Molecular Function of Cardiovascular disease, Department of Cardiology, Tianjin Institute of Cardiology, Second Hospital of Tianjin Medical University, Tianjin 300211, People’s Republic of China; 2 Second Department of Cardiology, Laboratory of Cardiac Electrophysiology, “Evangelismos” General Hospital of Athens, Athens, Greece; 3 First Department of Cardiology, University of Ioannina Medical School, Ioannina, Greece; 4 Lankenau Institute for Medical Research and Lankenau Medical Center, Wynnewood, Pennsylvania; Jefferson Medical College, Philadelphia, Pennsylvania, USA; 5 The First Affiliated Hospital, Medical School of Xi’an Jiaotong University, Xi’an, China

## Abstract

The aim of this of this meta-analysis was to examine the potential association between certain early repolarization (ER) characteristics and ventricular tachyarrhythmias (VTAs) in patients with acute myocardial infarction (AMI). We searched PubMed, Embase and Web of Science databases for records published until December 2014. Of the 658 initially identified records, 7 studies with a total of 1,565 patients (299 with ER and 1,266 without ER) were finally analyzed. Overall, patients with ER displayed a higher risk of VTAs following AMI compared to patients without ER [odds ratio (OR): 3.75, 95% CI: 2.62–5.37, p < 0.00001]. Subgroup analyses showed that the diagnosis of ER prior to AMI onset is a better predictor of VTAs (OR: 5.70, p < 0.00001) compared to those diagnosed after AMI onset (OR: 2.60, p = 0.00001). Remarkably, a notching morphology was a significant predictor of VTAs compared to slurring morphology (OR: 3.85, p = 0.002). Finally, an inferior ER location (OR: 8.85, p < 0.00001) was significantly associated with increased risk of VTAs in AMI patients. In conclusion, our meta-analysis suggests that ER pattern is associated with greater risk of VTAs in patients with AMI. A notched ER pattern located in inferior leads confers the highest risk for VTAs in AMI.

The early repolarization (ER) is characterized by an elevation ≥0.1 mV of the QRS-ST junction (J-point) in at least 2 inferior or lateral leads on 12-lead electrocardiography (ECG). The J-point elevation may manifest as QRS slurring or notching. Recently, population-based studies linked J-point elevation to idiopathic ventricular fibrillation (VF) and sudden cardiac death (SCD) in subjects with no structural heart disease^1^,^2^,^3^,^4^,^5^ and to ventricular tachyarrhythmias (VTAs) in patients who have chronic coronary artery disease^6^. Several studies suggested that the presence of ER pattern is associated with occurrence of VTAs during acute myocardial infarction (AMI)^7^,^8^,^9^,^10^,^11^,^12^,^13^. However, the role of certain ER pattern characteristics (diagnosis of ER before or after AMI onset, J-point morphology, and lead distribution) in predicting the occurrence of VTAs in patients with AMI needs further investigation. Therefore, we conducted a systematic review and meta-analysis to examine the predictive value of detailed ER pattern characteristics on the occurrence of VTAs in the setting of AMI.

## Methods

Meta-analyses of observational studies present particular challenges because of inherent biases and differences in study designs. Consequently, we performed this analysis according to the Strengthening the Reporting of Observational Studies in Epidemiology (STROBE) statement^14^.

### Inclusion criteria

We included observational studies with a primary objective to analyze the potential association between ER pattern and the occurrence of VTAs in the setting of AMI. Titles and abstracts of all articles were evaluated and rejected after initial screening according to the following inclusion criteria: (1) published in the English language; (2) human subjects; (3) the study design was a case–control study, prospective cohort study or retrospective cohort study. Individual case reports, review articles and editorials were excluded; (4) assessed ER and documented VTAs in the setting of AMI; (5) clearly defined endpoint events, such as non-sustained ventricular tachycardia (VT), sustained VT and ventricular fibrillation (VF); (6) reporting the odds ratios (ORs) or hazard ratios (HRs) and the corresponding 95% confidence intervals (CIs) or data necessary to calculate these for ER in AMI patients were reported.

### Search strategy

A systematic literature search was performed by two investigators (Z. Z. and Y. Y.) using the online databases of PubMed, Embase and Web of Science in order to identify relevant studies from 1971 to December 2014. We used the following keywords: “early repolarization” and “myocardial infarction”. Titles and abstracts as well as the reference lists from all the retrieved studies were manually checked independently by two investigators (Z. Z. and Y. Y.) to include potentially relevant records published until December 2014.

### Study selection

Two investigators (Z. Z. and Y. Y.) systematically extracted the data in pre-specified data forms. All potentially relevant reports were retrieved as complete manuscripts, and then we assessed them eligibility according to the inclusion criteria. Any disagreements or uncertainties between the two investigators were resolved through consensus after rechecking the source data and consultation with a third investigator (T. L.).

### Data extraction and Quality assessment

Two investigators (Z. Z. and Y. Y.) independently extracted the related data using a predefined form. In each primary study, we extracted all the adjusted and unadjusted (if the adjusted OR/HR were not applicable) OR/HR and the corresponding 95% CI or data necessary to calculate it in this way (categorical) to evaluate ER in predicting the risk of VTAs in the setting of AMI. The extracted data elements for each study included first author’s last name, publication year, the geographic location of study, study design, total number of participants, participants’ age and sex, co-morbidities, peak creatine kinase (CK) and CK-MB levels, medications, duration of follow-up, adjusted variables, ER definition, diagnosis of ER before or after AMI onset, end point events and number of incident cases. Bearing in mind that quality scoring in meta-analyses of observational studies is controversial, we systematically assessed the quality using a point score system which has been presented in our previous meta-analysis^15^.

### Statistical analysis

Pooled effect sizes were presented as ORs with 95% CIs. The HR value using multivariate Cox proportional hazards model in the primary study was directly considered as OR. The pooled effect estimates were evaluated using the inverse-variance weighting under fixed effects model. Therefore, we analyzed all the adjusted and unadjusted (if the adjusted OR/HR were not applicable) OR/HR and the corresponding 95% CI to evaluate ER in predicting the risk of VTAs in the setting of AMI. Statistical heterogeneity was assessed by using the I^2^ statistic, which was defined at least moderate heterogeneity as an I^2^ value of >50%^16^. A fixed effects model was used if no significant heterogeneity existed. We conducted fixed effects meta-analysis using the inverse variance method for pooling effect sizes, and random effects meta-analysis using the inverse variance heterogeneity method. Subgroup analyses regarding the diagnosis of ER in relation to AMI onset (before AMI ER and after AMI ER), the morphology of J-point (notching or slurring), the location of ER (inferior leads), the follow-up duration for detection of VTAs (≤48 hours or >48 hours) and if the studies solely included the patients with STEMI were additionally performed. Finally, we performed a sensitivity analysis by removing one study, where 29.4% of patients did not treated with PCI. We assessed publication bias by constructing a funnel plot. Two-tailed p values of <0.05 were considered statistically significant. All statistical analyses were performed with the use of Review Manager version 5.3.

## Results

**Figure 1:**
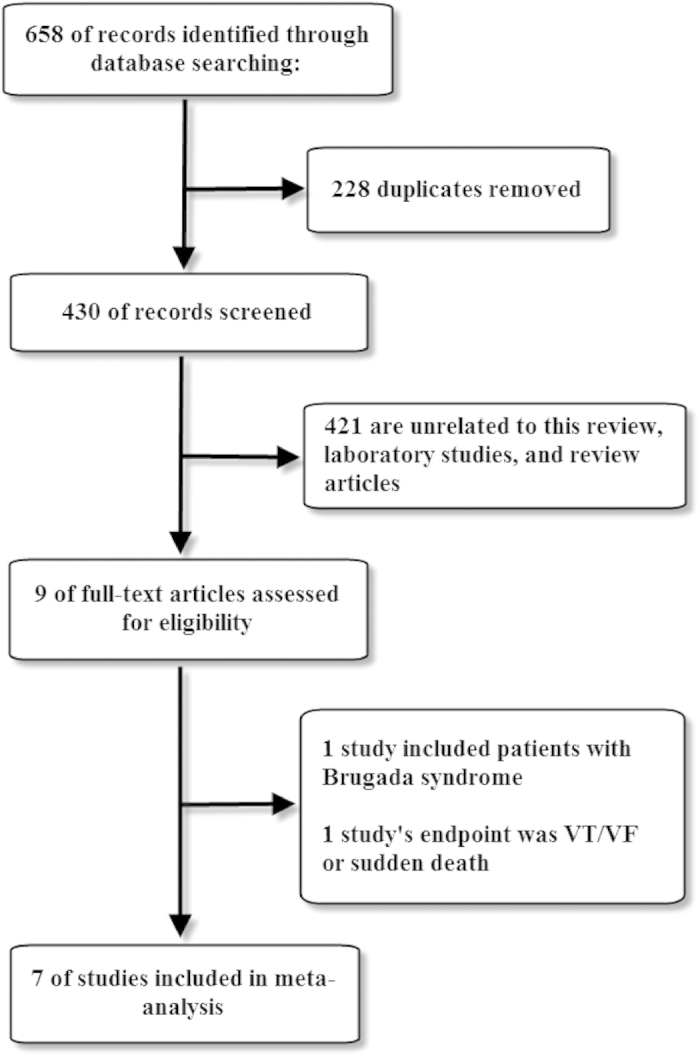
Flow diagram of the study selection process.

The study selection flow is summarized in Fig. 1. Following immediate exclusion of 228 replicated studies, 421 out of 430 studies were subsequently excluded for several reasons (review articles, laboratory studies, or irrelevant to the current analysis). After detailed review of the remaining 9 studies for eligibility, one^17^ was excluded because it included patients with Brugada syndrome while another one^18^ was excluded because its endpoint combined VTAs with SCD. Thus, 7 studies^7^,^8^,^9^,^10^,^11^,^12^,^13^ fulfilling the prespecified selection criteria were finally included in our analysis. Of the 1,565 patients who suffered AMI, 299 were classified in the ER(+) group and 1,266 in the ER(−) group. The 7 studies are shown in Table 1, whereas the patients’ characteristics are summarized in Table 2. Furthermore, the definition of AMI and methods of VTAs detection are provided in a supplement table.

**Figure 2:**
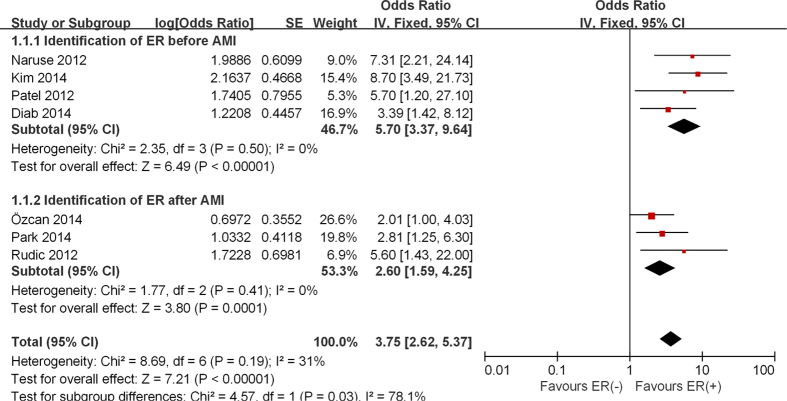
Forest plot demonstrating the association between ER pattern and the occurrence of VTAs in the patients with AMI including a subgroup analysis according to different diagnostic time of ER compare to AMI onset.

All studies consistently suggested that the ER pattern is associated with VTAs in the setting of AMI (in five^7^,^8^,^9^,^11^,^13^ after multivariate analysis and in two^10^,^12^ after univariate analysis). Overall, patients with ER exhibited nearly four times greater risk for VTAs compared to those without ER (OR 3.75, 95% CI: 2.62 to 5.37, p < 0.00001; Fig. 2). There was no significant heterogeneity among the individual studies (p = 0.19, I^2^ = 31%).

A subgroup analysis based on the time of ER diagnosis in relation to AMI occurrence was subsequently performed (Fig. 2). Four studies provided data on ECG recordings prior to AMI onset^7^,^8^,^12^,^13^. The pooled analysis of these studies^7^,^8^,^12^,^13^ (OR 5.70, 95% CI 3.37 to 9.64, p < 0.00001; Heterogeneity: p = 0.50, I^2^ = 0%) showed that the presence of ER before AMI doubles the risk of VTAs compared to those with ER recorded after AMI development^9^,^10^,^11^ (OR 2.60, 95% CI 1.59 to 4.25, p = 0.00001; Heterogeneity: p = 0.41, I^2^ = 0%).

**Figure 3:**
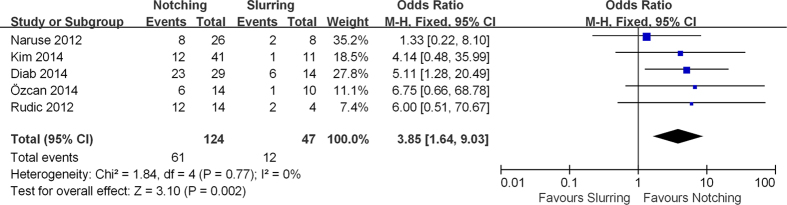
Forest plot comparing the impact of two different ER morphologies (notching vs. slurring pattern) on the occurrence of VTAs in the setting of AMI.

Subgroup analyses regarding the predictive value of J-point morphology^7^,^9^,^10^,^12^,^13^ and ER pattern location^7^,^9^,^12^ on the development of VTAs following AMI were performed as well (Table 3). We found that notching morphology (OR 8.32, 95% CI 4.92 to 14.09, p < 0.00001) and inferior ER location (OR 8.85, 95% CI 4.35 to 17.98, p < 0.00001) are both significantly associated with 8- and 9-fold increased risk for VTAs, respectively. Therefore, these characteristics seem to have further value in predicting VTAs occurrence in the setting of AMI. The slurring ER pattern (OR 1.53, 95% CI 0.692 to 3.38, p = 0.29) was not associated with an increased risk of VTAs following AMI. Furthermore, a direct comparison between the two different ER morphologies showed that a notched pattern increased the risk of VTAs up to 4-fold compared to the slurred pattern (OR 3.85, 95% CI 1.64 to 9.03, p = 0.002) (Fig. 3).

Two subgroup analyses regarding the follow-up duration for detection of VTAs (≤48 hours or >48 hours) and the type of AMI [ST-segment elevation myocardial infarction (STEMI) and non-ST-segment elevation myocardial infarction (NSTEMI)] were performed. As shown in Table 3, the ER pattern was associated with an increased risk of VTAs in both studies with short (≤48 hours)^7^,^12^,^13^ (OR 5.70, 95% CI 3.26 to 9.96, p < 0.00001) and long detection window (>48 hours)^8^,^9^,^10^,^11^ (OR 2.79, 95% CI 1.74 to 4.46, p < 0.0001). The analysis of the studies^8^,^10^,^11^,^13^ involving only patients with STEMI showed the ER pattern retains its predictive value (OR 2.73, 95% CI 1.77 to 4.21, p: <0.00001).

Finally, we performed a sensitivity analysis by removing the study by Diab *et al.*^13^, where a number of patients did not treated with PCI. The analysis from the remaining six studies did not show significant influence on heterogeneity across studies or overall results (OR = 3.82, 95% CI 2.58 to 5.67; I^2^ = 42%).

## Discussion

This comprehensive meta-analysis of seven observational studies demonstrated that patients with ER display a higher risk of VTAs occurrence in the setting of AMI. This association was consistently observed both in cohort and in case-control studies, as well as in diverse populations. Remarkably, subgroup analyses demonstrated that notching morphology and inferior ER location are significantly associated with VTAs in patients with AMI. The prognostic significance of ER pattern remained significant irrespective of the detection window used for VTAs.

A transmural voltage gradient caused by differences in the magnitude of Ito-mediated action potential notch between ventricular epicardium and endocardium is thought to be responsible for inscription of the ECG J-point. Transmural dispersion of repolarization may facilitate the induction of phase 2 reentry and provides the substrate for the development of VTAs^19^,^20^. Antzelevitch *et al.*^21^ have suggested that the J-wave is caused by a net outward increase in repolarizing current, which resulted from a decreased inward I_CaL_ or I_Na_ channel currents or an increased outward potassium currents mediated by I_to_, I_K-ATP_ and I_K-Ach_ channels. An association between the I_to_ density and the risk of VF in the setting of AMI has been previously demontstrated^22^,^23^. In this context, a higher density of the I_to_ current in the right ventricular epicardium than in the left ventricular epicardium may also explain the higher prevalence of primary VF seen in patients with an inferior AMI with right ventricular involvement compared to those with an anterior MI^24^,^25^. Acute regional myocardial ischemia may result in markedly heterogeneous loss of I_to_-mediated epicardial AP domes across the ischemic border, leading to phase 2 reentry to trigger VF^26^. In the present analysis we showed that an inferior ER pattern is associated with an increased risk of VTAs in acute AMI, an event possibly linked to the greater predominance of Ito current in the right ventricular epicardium. In our study, a notched ER pattern increased the risk of VTAs up to 4-fold compared to the slurred ER pattern in patients with AMI. A terminal QRS notching is considered more prevalent in malignant variants of ER in subjects with idiopathic ventricular fibrillation^27^.

Finally, we indicated that the presence of ER before AMI doubles the risk of VTAs compared to those with ER recorded after AMI. Thus, ER may aggravate the arrhythmogenic substrate in patients with AMI. Given that up to 5% of the general population displays an ER pattern^1^, this potential marker of arrhythmogenesis may facilitate the identification of patients at risk for experiencing life-threatening VTAs in the setting of an acute coronary syndrome.

### Study Limitations

The present meta-analysis has several limitations. First, given that the number of identified studies was relatively small and the available data limited, apart from J-point morphology (notching or slurring) and inferior leads ER location, we could not analyze other specific ER characteristics such as J-point amplitude, ER location in anterior leads and ST-segment morphology. Second, some of the ORs/HRs used in the present analysis were not derived from multivariate analyses. Third, the exact endpoints of the identified studies (non-sustained VT, sustained VT and VF) were not absolutely consistent, a fact that may indicate latent bias in this meta-analysis. Fourth, some important data such as troponin levels, and beta-blockers or other anti-arrhythmic drug use that may be related to VTAs incidence were not fully presented in our analysis due to the lack of evidence. Furthermore, we included patients with STEMI and non-STEMI. It should be acknowledged that these entities have different pathophysiological mechanisms and presumably different risk for VTAs. However, the subgroup analysis involving four studies with only STEMI patients showed similar results to the main analysis. Finally, we have observed little publication bias with a funnel plot analysis.

## Conclusions

In conclusion, our meta-analysis suggests a clear and strong association between ER and VTAs development in patients with AMI. A notched ER pattern located in inferior leads confers the highest risk for VTAs in AMI. Larger studies are needed in order to elucidate the prognostic significance of ER pattern in patients with AMI.

## Additional Information

**How to cite this article**: Zhang, Z. *et al.* Notching early repolarization pattern in inferior leads increases risk of ventricular tachyarrhythmias in patients with acute myocardial infarction: a meta-analysis. *Sci. Rep.* **5**, 15845; doi: 10.1038/srep15845 (2015).

## Supplementary Material

Supplementary Information

## Figures and Tables

**Table 1:** Characteristics of the seven studies included in meta-analysis.

Firstauthor(Year)	Location	Patients(n)	ER(+)n(%)	Studydesign	ER definition	The variables ofmultivariate model	Endpoints	Follow-up	Qualityscore
Identification of ER before AMI									
Naruse *et al.*^7^ (2012)	Japan	220	34 (16)	Cohort	An elevation of the QRS-ST junction of >0.1 mV from baseline in at least 2 inferior or lateral leads, manifested as QRS slurring or notching	Age per year, male sex, time from the symptom onset to emergency room of <180 minutes, Killip class >1, peak CK levels >3000 U/L, No. of diseased coronary arteries >1, STEMI, hypertension, diabetes mellitus, smoking	Sustained VF	48 hours	9
Kim *et al.*^12^(2014)	Korea	296	52 (17.6)	Cohort	J point elevations manifested through QRS notching or slurring for at least 1 mm (0.1 mV) above the baseline in at least two consecutive inferior or lateral leads	Unadjusted	VF	48 hours	9
Patel *et al.*^8^(2012)	America	100	30 (30)	Case-control	Notching, slurring, or J-point elevation ≥0.1 mV above baseline in ≥2 contiguous inferior, lateral, or anterior leads	LVEF	VTAs(Non-sustained VT, Sustained VT, VF)	72 hours	9
Diab *et al.*^13^(2014)	Egypt	102	43 (42.2)	Case-control	≥1 mm J point elevation with or without ST segment elevation.	LVEF, QTc, and QTd	VTAs(Sustained VT, VF)	48 hours	8
Identification of ER after AMI									
Özcan *et al.*^10^ (2014)	Turkey	521	61 (11.7)	Cohort	Elevation of the J-point (QRS–ST Junction) above 0.1 mV relative to QRS onset in two or more inferior leads (DII–DIII-aVF), limb lateral leads (DI-aVL), or left precordial leads (V4–V6)	Unadjusted	VTAs(Sustained VT, VF)	30 days	8
Park *et al.*^11^ (2014)	Korea	266	76 (28.6)	Cohort	J-point elevation ≥0.1 mV and “notching” and “slurring” of the terminal part of the QRS complex in at least 2 lateral or inferior leads.	Inferior MI, E/E′, LVEF (<45%)	VTAs(Non-sustained VT, Sustained VT, VF)	6.7 ± 4.5 days	8
Rudic *et al.*^9^ (2012)	Germany	60	18 (30)	Case-control	J-point elevation ≥0.1 mV and “notching” and “slurring” of the terminal part of the QRS complex in at least 2 lateral or inferior leads.	LVEF and QTc	VF	During hospitalization	7

ER = early repolarization; STEMI = ST-elevation myocardial infarction; AMI = acute myocardial infarction; CK = creatine kinase; LVEF = left ventricular ejection fraction; VF = ventricular fibrillation; VT = ventricular tachycardia; VTAs = ventricular tachyarrhythmias.

**Table 2:** Patient characteristics of the seven included studies.

FirstAuthor	Patients(n)	Male(%)	Mean age(years)	HTNn (%)	DMn (%)	Hyperlipidemian (%)	Smokingn (%)	LVEF(%)	Peak CK orCK-MB levels(U/L)	Medication			PCIn (%)	Anterior MIn (%)	STEMIn (%)
										Statinsn (%)	β-Blockersn (%)	AADsn (%)			
Naruse *et al.*^7^	220	163(74)	69 ± 11	153(70)	82(37)	107(49)	110(50)	NA	2315 ± 2101 (CK)	NA	NA	NA	220(100)	NA	175(80)
Kim *et al.*^12^	296	223(75.3)	61.23 ± 13.41	155(52.4)	64(21.6)	NA	176(59.5)	46.57 ± 8.48	2654.87 ± 4103 (CK)	NA	NA	NA	296(100)	NA	193(65.2)
Patel *et al.*^8^	100	60(60)	65.5	77(77)	30(30)	NA	59(59)	44.5	160.1(CK-MB)	46(46)	41(41)	NA	100(100)	49(49)	100(100)
Diab *et al.*^13^	102	102(100)	48.4	36(35.3)	39(38.2)	NA	34(33.3)	42.3	NA	NA	NA	NA	72(70.6)	62(60.8)	102(100)
Özcan *et al.*^10^	521	433(83.1)	57.2	217(41.7)	95(18.2)	75(14.4)	360(69.1)	45.7	131.1(CK-MB)	NA	NA	NA	521(100)	269(51.6)	521(100)
Park *et al.*^11^	266	217(81.6)	61.6 ± 12.7	130(48.9)	72(27.1)	162(60.9)	NA	51.2 ± 11.8	NA	NA	NA	0(0)	266(100)	118(44.4)	266(100)
Rudic *et al.*^9^	60	48(80)	61.8 ± 13.1	33(55)	16(27)	21(35)	NA	NA	NA	NA	NA	0(0)	60(100)	17(28)	33(55)

HTN = hypertension; DM = diabetes mellitus; LVEF = left ventricular ejection fraction; CK = creatine kinase; CK-MB = creatine kinase-MB; AADs = antiarrhythmic drugs; PCI = percutaneous coronary intervention; MI = myocardial infarction; STEMI = ST-segment elevation myocardial infarction; NA = not applicable.

**Table 3:** Subgroup analyses of the association between ER and VTAs during AMI.

Subgroup	Study	Number ofstudies	Heterogeneity		Meta-analysis		
			I^2^	p-Value	OR	95% CI	p-Value
Identification time of ER	Before AMI	4	0%	0.50	5.70	3.37–9.64	<0.00001
	After AMI	3	0%	0.41	2.60	1.59–4.25	0.0001
Morphology	Notching	5	0%	0.77	8.32	4.92–14.09	<0.00001
	Slurring	5	0%	0.46	1.53	0.69–3.38	0.29
Distribution	Inferior leads	3	16%	0.30	8.85	4.35–17.98	<0.00001
Follow-up duration	≤48 hours	3	15%	0.31	5.70	3.26–9.96	<0.00001
	>48 hours	4	0%	0.45	2.79	1.74–4.46	<0.0001
All patients with STEMI	Yes	4	0%	0.61	2.73	1.77–4.21	<0.00001
	No	3	0%	0.87	7.51	3.95–14.27	<0.00001

ER = early repolarization; AMI = acute myocardial infarction; VF = ventricular fibrillation; VTAs = ventricular tachy-arrhythmias; STEMI = ST-segment elevation myocardial infarction; OR = odds ratio; CI = confidence interval.
