# Supplementary material for: Notching early repolarization pattern in inferior leads increases risk of ventricular tachyarrhythmias in patients with acute myocardial infarction: a meta-analysis
Source: Sci Rep. 2015 Nov 2;5:15845. doi: 10.1038/srep15845 (PMC4629141; doi:10.1038/srep15845)
Supplement: Supplementary Information [file srep15845-s1.doc]

***Notching early repolarization pattern in inferior leads increases risk of ventricular tachyarrhythmias in patients with acute myocardial infarction: a meta-analysis***

***Zhiwei Zhang^1^, Konstantinos P. Letsas^2^, Yajuan Yang^1^, Panagiotis Korantzopoulos^3^, Guangping Li^1^, Gan-Xin Yan^4,5^, Tong Liu^1^****

*1 Tianjin Key Laboratory of Ionic-Molecular Function of Cardiovascular disease, Department of Cardiology, Tianjin Institute of Cardiology, Second Hospital of Tianjin Medical University, Tianjin 300211, People’s Republic of China*

*2 Second Department of Cardiology, Laboratory of Cardiac Electrophysiology, “Evangelismos” General Hospital of Athens, Athens, Greece.*

*3 First Department of Cardiology, University of Ioannina Medical School, Ioannina, Greece*

*4 Lankenau Institute for Medical Research and Lankenau Medical Center, Wynnewood, Pennsylvania; Jefferson Medical College, Philadelphia, Pennsylvania, USA*

*5 The First Affiliated Hospital, Medical School of Xi'an Jiaotong University, Xi'an, China.*

**Key Words:** Early repolarization; ventricular tachyarrhythmias; acute myocardial infarction; meta-analysis

**Word Count (Including References): 3207**

*Corresponding author: Associate Professor Tong Liu, Tianjin Key Laboratory of Ionic-Molecular Function of Cardiovascular disease, Department of Cardiology, Tianjin Institute of Cardiology, Second Hospital of Tianjin Medical University, No. 23 Pingjiang Road, Hexi District, Tianjin 300211, People’s Republic of China.

Tel.: +86-22-88328617; Fax: +86-22-28261158

E-mail: liutongdoc@126.com

Supplement table：Acute myocardial infaction definition and methods of ventricular arrhythmias detection in seven included studies.

| First Author ^Ref^ | AMI definition | Methods of VTAs detection | Troponin (μg/L) |
| --- | --- | --- | --- |
| Naruse et al^7^ | Within 24 hours of the onset of the symptoms associated with an AMI | NA | NA |
| Kim et al^12^ | Within 48 hours from the onset of chest pain | NA | NA |
| Patel et al^8^ | The first 72 hours of the index hospitalization | Chart review with an accompanying rhythm strip or ECG of the VTA episode | 29.8 |
| Diab et al^13^ | During the first 48 hours of myocardial insult | Continuous ECG monitoring during the first 48 hours. Any rhythm disturbance was documented with either 12-lead ECG or rhythm strip | NA |
| Özcan et al^10^ | Within 12 hours of the onset of symptoms | NA | NA |
| Park et al^11^ | Patients underwent primary percutaneous coronary intervention on admission | 12-lead ECG | NA |
| Rudic et al^9^ | Patients underwent primary percutaneous coronary intervention on admission | 12-lead ECG | NA |

AMI = acute myocardial infarction; VTAs = ventricular tachyarrhythmias; NA = not applicable; ECG = electrocardiogram
